# Evaluating peripartum calcium administration strategies to improve sow farrowing performance and piglet livability

**DOI:** 10.1093/tas/txaf142

**Published:** 2025-10-23

**Authors:** Abigail K Jenkins, Sierra M Collier, Sara Virdis, Olivia J Cataldo, Mike D Tokach, Joel M DeRouchey, Jason C Woodworth, Katelyn N Gaffield, Jordan T Gebhardt, Robert D Goodband, Kyle F Coble, Paul J Corns, Jimmy Karl, Tag Bradley, Jose A Soto, Andrew Bents

**Affiliations:** Department of Animal Sciences and Industry, College of Agriculture, Kansas State University, Manhattan, KS 66506-0201, United States; Department of Animal Sciences and Industry, College of Agriculture, Kansas State University, Manhattan, KS 66506-0201, United States; Department of Animal Sciences and Industry, College of Agriculture, Kansas State University, Manhattan, KS 66506-0201, United States; Department of Animal Sciences and Industry, College of Agriculture, Kansas State University, Manhattan, KS 66506-0201, United States; Department of Animal Sciences and Industry, College of Agriculture, Kansas State University, Manhattan, KS 66506-0201, United States; Department of Animal Sciences and Industry, College of Agriculture, Kansas State University, Manhattan, KS 66506-0201, United States; Department of Animal Sciences and Industry, College of Agriculture, Kansas State University, Manhattan, KS 66506-0201, United States; Department of Animal Sciences and Industry, College of Agriculture, Kansas State University, Manhattan, KS 66506-0201, United States; Department of Diagnostic Medicine/Pathobiology, College of Veterinary Medicine, Kansas State University, Manhattan, KS 66506-0201, United States; Department of Animal Sciences and Industry, College of Agriculture, Kansas State University, Manhattan, KS 66506-0201, United States; JBS Live Pork, Greeley, CO 80634-9039, United States; JBS Live Pork, Greeley, CO 80634-9039, United States; JBS Live Pork, Greeley, CO 80634-9039, United States; JBS Live Pork, Greeley, CO 80634-9039, United States; Alltech, Nicholasville, KY 40356-8700, United States; Alltech, Nicholasville, KY 40356-8700, United States

**Keywords:** calcium chloride, calcium gluconate, farrowing, sow, stillbirths

## Abstract

A total of 933 sows were used to evaluate peripartum calcium administration protocols on sow farrowing performance and piglet livability. Sows were blocked by parity and average stillbirths in previous parities then allotted to 1 of 3 treatments with 310 to 312 replications. Treatments included: (i) Control with sows receiving no intervention; (ii) 25 g of a calcium chloride-based product (CaCl_2_) top-dressed daily each morning from approximately d 112 of gestation until farrowing; or (iii) calcium gluconate injection (CaG) administered to primiparous and multiparous sows (15 or 20 mL injection, respectively), at the time that a sow was classified as “at-risk” defined by the sow, in the current parturition, having more than 16 pigs, longer than 1 h since the birth of the last pig, 2 or more stillbirths, or farrowing duration exceeding 4 h. On a subset of sows farrowing duration, sow blood metabolites, sow urine pH, and pig immunocrit were analyzed. Parity category (P1, P2 to P4, or P5+), treatment, and their interaction were fixed effects while average past stillbirth category (<0.5, ≥0.5 and ≤1, or >1 stillborn/litter) was a random effect. There were no differences in total born, percentage born alive, or percentage stillborn between treatments; however, when at-risk sows were compared, administration of CaG decreased stillbirths and increased percentage of pigs born alive (*P* ≤ 0.006). There was an interaction between Ca protocol and parity category for birth to cross-foster mortality (*P* = 0.035) where mortality was lowest in P1 Control sows (*P* < 0.05) compared to all other treatment × parity category combinations except for P1 CaG sows which were intermediate. When considering all sows, sows fed CaCl_2_ had increased blood Cl and ionized Ca (*P* < 0.001) compared to Control or CaG sows. Sows provided CaG had increased blood glucose (*P* = 0.026) compared to Control and CaCl_2_ sows. Sows given CaCl_2_ or CaG had decreased urine pH (*P* = 0.001) compared to Control sows. Pig immunocrit ratios tended (*P* = 0.068) to differ due to Ca protocol, with CaG offspring having a numerical increase compared to other treatments. In conclusion, in the overall population, top-dressing CaCl_2_ before farrowing or injecting CaG peripartum altered sow metabolites but did not influence farrowing performance. However, when comparing at-risk sows, administration of CaG decreased stillbirths and increased percentage of pigs born alive.

## Teaser

Calcium gluconate injection decreased stillbirths and increased the percentage of pigs born alive in sows identified as at-risk for greater stillbirth incidence. Providing a calcium chloride top-dress did not influence stillbirths.

## Introduction

Among US swine farms with the lowest pigs weaned per mated female per year, stillbirths account for 8.8% of total pigs born (MetaFarms 2024). This high rate of stillbirths represents a significant economic loss for producers by reducing the number of marketable pigs and overall system productivity. Known risk factors associated with increased stillbirth incidence include older parity, prolonged farrowing duration, large litter sizes, late birth order, and lack of farrowing supervision (Le Cozler et al. 2002; Adi et al. 2024). A broad range of factors, such as nutritional management, parturition induction protocols, farrowing supervision practices, pharmacological and manual interventions, environmental conditions, and stockmanship, have been examined for their influence on the incidence of stillborn pigs (Vanderhaeghe et al. 2013). Recent findings by Craig et al. (2024) highlight a physiological contributor, showing increased stillbirth rates in sows with serum Ca level below a threshold of 2.5 mmol/L before farrowing. Calcium plays a critical role in smooth muscle contraction, including uterine contraction (Uehata et al. 1997), which may help explain the increased incidence of dystocia and stillbirths in sows with low serum Ca levels.

Calcium chloride (CaCl_2_) has been supplemented to sows in the feed during the transition period from entry into the farrowing house until parturition for its potential to reduce stillbirth incidence (Elrod et al. 2015; Bents and Soto 2023; González-Sánchez et al. 2023). In addition to supplying Ca, CaCl_2_ serves to lower the dietary cation-anion difference (DCAD), which enhances Ca absorption from the small intestine and mobilization from bone in dairy cattle (Abu Damir et al. 1994). While there is less knowledge about Ca homeostasis in sows around parturition, DeRouchey et al. (2003) observed increased ionized Ca when DCAD was reduced. Previous studies on CaCl_2_ supplementation in the feed have reported variable effects on stillbirth rates (González-Sánchez et al. 2023; Craig et al. 2024; Ruampatana et al. 2025). Another Ca supplement used in US swine production systems to support farrowing performance is injectable Ca gluconate (CaG). This compound is commonly administered in dairy cattle to treat hypocalcemia which is a condition triggered by the sudden increase in Ca demand before parturition due to the rapid increase in milk production (Oetzel 2022). Although no controlled research trials have evaluated the use of CaG injections during farrowing in sows, case studies suggest it may alleviate dystocia symptoms (Durrell 1942; Chutia et al. 2018; Reshma et al. 2020). Therefore, the objective of this trial was to evaluate 2 peripartum Ca administration protocols on piglet livability, sow performance before cross-fostering, and sow and piglet blood parameters. Our hypothesis was that increasing the amount of Ca available during parturition would improve uterine contractions, as evidenced by reduced stillbirths, shortened farrowing durations, and reduced early pig mortality.

## Materials and methods

### General

The protocol for this experiment was approved by the Kansas State University Institutional Animal Care and Use Committee (IACUC #4915.02). This study was conducted on a commercial sow farm in northwest Texas. Sows were individually housed in an environmentally controlled and mechanically ventilated barn. Farrowing stalls were equipped with a dry *ad libitum* sow feed dispenser (SowMax, HogSlat, Newton Grove, NC, United States) with a dry feeder and a nipple waterer placed at sow shoulder height. A nipple waterer at the base of the farrowing stall and a heat lamp was included in every farrowing stall for pig comfort.

### Animals and treatment structure

A total of 933 sows (average parity 3.3, Line 1050, PIC, Hendersonville, TN, United States) and their litters were used from May to August 2024. On approximately d 112 of gestation, sows were weighed and moved into the farrowing house from the gestation facility. Sows were blocked by parity and past average stillbirths and assigned to 1 of 3 Ca administration protocols. Parity categories were: first parity (*n* = 194), parity 2 to 4 (*n* = 489), and parities 5 and greater (*n* = 250). Average past stillbirth categories of multiparous sows (*n* = 739) were: <0.5 (*n* = 326), 0.5 ≥ x ≤ 1.0 (*n* = 278), and >1.0 (*n* = 135). Within block, sows were randomly assigned to treatment to equalize parity and average past stillbirths. Treatments included: (i) sows which received no intervention (Control); (ii) 25 g of a calcium chloride-based product (CaCl_2_; TRIAD, Alltech, Inc., Nicholasville, KY, United States) top-dressed daily at the morning feeding from the time of entry (approximately d 112 of gestation) until the sow farrowed; or (iii) calcium gluconate injection protocol (CaG; VetOne; Boise, ID, United States) where primiparous and multiparous sows received a 15 or 20 mL, respectively, injection intramuscularly in the neck at the time a sow met an “at-risk” criterion, defined as being at increased risk of high stillbirth incidence. At-risk criteria included sows that, during the current parturition, had more than 16 pigs, went longer than 1 h since the birth of the last pig, had 2 or more stillbirths in the litter, or had a total farrowing duration that exceeded 4 h. Sows allotted to the calcium gluconate treatment that were deemed at-risk sows (*n* = 144) received the calcium gluconate in 2 equal injections, one on each side of the neck, using a 16-gauge × 3.8-cm needle. On average, the completion of farrowing for at-risk sows occurred 75 min after the calcium gluconate injection. If more than 1 h passed after CaG administration without expulsion of a pig or placenta, a second dose was administered. In total, 8 CaG sows received 2 doses. Sows received the CaCl_2_ top-dress which is a proprietary blend of CaCl_2_, *Yucca schidigera* extract, and flavors encapsulated in a lipid matrix for an average of 4.8 ± 0.14 d before farrowing. All sows were fed approximately 1.8 kg/d (0.9 kg each morning and afternoon) from d 112 of gestation until farrowing of a common sorghum-soybean meal-based lactation diet formulated to 1.05% standardized ileal digestible Lys and contained 0.88% Ca and 0.48% P. All sows were hand-fed before and on the day of farrowing and had *ad libitum* access to a common lactation diet after farrowing.

Farrowing was monitored from 6:00 a.m. until 3:00 p.m. daily. All sows, regardless of farrowing timing, were included in the analysis of this study. For each sow, litter characteristics (liveborn, stillborn, and born mummified) were recorded on a sow card by a member of the farm farrowing team during each walk through the farrowing house (approximately every 15 min). If it had been longer than 30 min since the birth of the last pig or the sow appeared to be in distress, farrowing assistance (sleeving) occurred and if a pig was present in the birth canal, it was manually removed. Any instances of farrowing assistance were recorded on the sow card, regardless of whether a pig was removed or not. Oxytocin (20 IU/mL; VetOne; Boise, ID, United States) was used sparingly in this trial and only after agreed upon by 2 independent parties of the research team. A total of 8 sows received oxytocin during this trial, with 4 sows from the CaG treatment and 4 sows from the CaCl_2_ treatment. Sows were only induced to farrow if gestation length reached 118 d. A total of 28 sows were induced using prostaglandin (Lutalyse, Zoetis Inc., Kalamazoo, MI, United States), with 13 sows from the control treatment, 8 sows from the CaCl_2_ treatment, and 7 sows from the CaG treatment.

Any pig mortalities that occurred before cross-fostering (at approximately 24 h of age) were recorded. The cause of mortality was also recorded. Insemination date after weaning was recorded (CloudFarms system, BASF, Florham Park, NJ, United States) and used to calculate the wean-to-estrus interval (WEI) for each sow that remained in the herd after weaning.

### Blood and urine analyses

On a subset of sows (*n* = 74/treatment), more extensive data collection was performed, including sow blood metabolites, urine pH, and piglet immunocrit ratios. Farrowing duration was recorded for these same sows, as well as for additional sows that became available. Specifically, farrowing duration was collected on 79 control sows, 84 CaCl_2_ sows, and 85 CaG sows. The additional sows were included when they farrowed during working hours and research personnel were present to collect the data. The beginning of farrowing was denoted as the birth of the first pig and the end of farrowing was retrospectively denoted as the birth of the last pig. The first 5 pigs born were marked (All-Weather Paint Stik, LA-CO Industries, Inc., Elk Grove Village, IL, United States) for split-suckling purposes. Split suckling occurred if there were more than 14 liveborn pigs in a litter. The first 5 pigs born were isolated underneath the heat lamp for no longer than 90 min and then they were reintroduced to the litter. Within 4 h of the end of farrowing, blood (1 mL) was collected from a prominent auricular vein of the sow using a 21-gauge × 1.9-cm needle. Before collection, the site was cleaned with 75% alcohol. The samples were kept in a 1 mL vacutainer tube (MiniCollect Lithium Heparin, Greiner Bio-One, Kremsmünster, Austria) for a maximum of 10 min before analysis (iSTAT CHEM8+ cartridge, Zoetis, Parsippany, NJ, United States). Sow blood metabolites including ionized Ca, glucose, blood urea nitrogen, Na, K, Cl, total carbon dioxide, anion gap, creatinine, hematocrit, and hemoglobin were determined. Within 6 h of the end of farrowing, sow urine was collected via the method described by Nickel et al. (2017). Urine was extracted into 50 mL specimen cups and immediately analyzed for pH using a portable pH meter (Fisherbrand accumet AP115 Portable pH Meter, Fisher Scientific, Waltham, MA, United States). At approximately 24 h after the beginning of farrowing, blood was taken for determination of immunocrit ratio from one of the first 5 pigs born, visually selecting the pig closest to the average birth weight of the litter. Immunocrit ratio was determined according to Vallet et al., (2013) and was utilized as an estimation of colostrum consumption. Because sows were assigned to treatment before farrowing, there were also sows assigned to the CaG treatment that were not later classified as at-risk sows and, thus, did not receive an injection of CaG. Within the CaG treatment, only sows that met the criteria for the injection were collected for sow blood metabolite, sow urine pH, and pig immunocrit analysis.

### Statistical analysis

Data were analyzed using the lme4 package of R (Version 4.0.0, R Foundation for Statistical Computing, Vienna, Austria) as a generalized randomized complete block design. Blocking structure accounted for parity and average past stillbirth record. Sow served as the experimental unit. Treatment, parity category, and their interaction were included as fixed effects. Past stillbirth category was included in the model as a random effect. Count data were analyzed using a negative binomial distribution using a log link function. Proportion data, including birth to cross-foster mortality and litter characteristics, were analyzed using a binomial distribution using a logit link function. The percentage of sows that were sleeved was analyzed using a binary distribution. Differences were considered significant at *P* ≤ 0.05 and marginally significant at 0.05 < *P* ≤ 0.10. All treatment × parity interactions were *P* > 0.05 unless otherwise indicated.

Sows were also categorized into 2 subsets: at-risk sows (*n* = 411) or other sows (*n* = 522) across the 3 treatments for additional statistical analysis. At-risk sows were those that met the criteria to receive Ca gluconate injection (sows with >16 pigs, >1 h since the last birth, ≥2 stillbirths, or farrowing lasting > 4 h) and farrowed during hours when personnel were present (between the hours of 6:00 a.m. until 3:00 p.m.). Sows assigned to the CaG treatment that met these criteria received an injection, whereas at-risk sows in the control and CaCl_2_ treatments met the criteria but did not receive an injection. The most common criteria leading to a sow being classified as at-risk were litters with >16 pigs and a gap of more than 1 h since the last pig was born. Because sows were assigned to treatment before farrowing, there were also sows assigned to the CaG treatment that were not later classified as at-risk sows and, thus, did not receive an injection of CaG. Other sows included those that farrowed during working hours (6:00 a.m. until 3:00 p.m.) but did not meet the criteria for a CaG injection, as well as those that farrowed overnight when farrowing intervention was unavailable. The same statistical analyses were performed with the same statistical models, but within risk group for the 3 treatments.

## Results

### Overall population

There was a treatment × parity interaction (*P* = 0.019) for the proportion of mummies in the litter however, means did not separate based on the Tukey analysis (Table S1). There was a treatment × parity interaction (*P* = 0.035) in birth to cross-foster mortality where parity 1 control sows had the lowest birth to cross-foster mortality compared to all other parity by treatment categories (*P* < 0.05) except for parity 1 CaG sows which were intermediate.

In the overall population, there was no difference in total born, the proportion of the litter born alive, or the proportion of stillbirths in the litter due to Ca administration protocol (Table 1). There were no differences in the percentage of sows sleeved, the number of times that sows needed to be sleeved, or farrowing duration due to Ca administration protocol. Birth to cross-foster mortality was increased (*P* < 0.001) in CaCl_2_ sows compared to control sows with CaG sows intermediate. Calcium administration protocol did not affect WEI.

**Table 1.** Effect of farrowing calcium protocol on sow performance.^1^

	Farrowing protocol^2^				*P* =		
	None	Calcium chloride	Calcium gluconate	SEM	Treatment × parity	Treatment	Parity
**Count, *n***	311	312	310	—	—	—	—
**Sow entry BW, kg**	269.0	269.9	268.9	2.45	0.478	0.891	<0.001
**Gestation length, d**	116.1	116.1	116.0	0.08	0.310	0.679	0.362
**Litter characteristics**							
** Total born, *n***	16.7	16.7	16.9	0.25	0.907	0.963	0.600
** Born alive, %**	90.8	90.1	91.4	0.88	0.190	0.145	<0.001
** Stillborn, %**	6.7	6.7	6.1	0.87	0.462	0.226	<0.001
** Mummy, %**	2.5	3.1	2.6	0.26	0.019	0.035	0.228
**Farrowing characteristics**							
** Percentage of sows sleeved**	45.0	48.7	51.3	3.32	0.278	0.381	<0.001
** Number of times sleeved**	1.1	1.1	1.3	0.39	0.970	0.901	0.458
** Farrowing duration, min^3^**	294.6	294.2	323.7	15.07	0.951	0.194	0.350
**Birth-cross foster mortality, %**	5.9^b^	7.9^a^	7.1^ab^	0.42	0.035	<0.001	<0.001
**Wean-to-estrus interval, d**	4.8	5.2	4.8	0.28	0.256	0.349	0.053

a-c Means within row with different superscripts differ (*P* < 0.05).

1 A total of 933 mixed-parity sows (Line 1050, PIC, Hendersonville TN) and litters were used from the time of entry into the farrowing house (approximately d 112 of gestation) until cross-fostering at approximately 24 h after farrowing.

2 Farrowing protocol consisted of a control with sows receiving no intervention (None), 25 g of a Ca chloride based product (TRIAD, Alltech, Lexington, KY) top-dressed on the morning feeding of each sow from entry into the farrowing house until each sow farrowed (Calcium chloride), and a Ca gluconate protocol in which primiparous sows received a 15 mL injection and multiparous sows received a 20 mL injection of Ca gluconate if a litter had more than 16 pigs, it had been longer than 1 h since the birth of the last pig, the litter had 2 or more stillbirths, or farrowing duration exceeded 4 h (Calcium gluconate).

3 Farrowing duration was collected on 79 Control sows, 84 Calcium chloride sows, and 85 Calcium gluconate sows.

Anion gap, blood urea nitrogen, creatinine, K, and Na in blood did not differ based on Ca administration protocol (Table 2). Chloride concentration was greater (*P* < 0.001) in the blood from CaCl_2_ sows compared to control and CaG sows. Blood glucose concentrations were greater (*P* = 0.026) in CaG sows compared to control and CaCl_2_ sows. There was a tendency (*P* ≤ 0.067) for a difference in hematocrit and hemoglobin concentrations based on Ca administration protocol where CaCl_2_ sows had a numeric increase in both criteria, followed by CaG sows, and then by control sows. Circulating ionized calcium (iCa) concentrations were increased (*P* < 0.001) in CaCl_2_ sows compared to both CaG and control sows. Total carbon dioxide (tCO_2_) concentration in blood was decreased (*P* < 0.001) in CaCl_2_ sows compared to control sows with CaG sows intermediate. Urine pH was decreased (*P* = 0.001) in CaG and CaCl_2_ treatment sows compared to control sows. There was a tendency for a difference (*P* = 0.068) in pig immunocrit ratio where pigs from CaG sows had a numeric increase in immunocrit ratio compared to pigs from control sows.

**Table 2.** Effect of farrowing calcium protocol on sow blood and urine characteristics and piglet immunocrit ratio.^1^

	Farrowing protocol^2^				*P* =		
	None	Calcium chloride	Calcium gluconate	SEM	Treatment × parity	Treatment	Parity
**Count, *n***	74	74	74	—	—	—	—
**Blood characteristics**							
** Anion gap, mmol/L**	17.6	18.0	18.2	0.25	0.761	0.236	0.561
** Blood urea nitrogen, mg/dL**	12.6	13.0	13.6	0.71	0.232	0.401	0.405
** Chloride, mmol/L**	102.9^b^	104.6^a^	103.0^b^	0.39	0.973	<0.001	0.161
** Creatinine mg/dL**	3.1	3.2	3.2	0.07	0.643	0.944	0.001
** Glucose, mmol/L**	96.5^b^	96.4^b^	102.8^a^	2.46	0.765	0.026	0.059
** Hematocrit, % PCV**	31.0	32.6	31.7	0.56	0.510	0.060	0.455
** Hemoglobin, g/dL**	10.5	11.1	10.8	0.19	0.495	0.067	0.455
** Ionized Calcium, mmol/L**	1.23^b^	1.29^a^	1.24^b^	0.016	0.212	<0.001	0.001
** Potassium, mmol/L**	4.2	4.2	4.1	0.05	0.587	0.365	0.058
** Sodium, mmol/L**	142.6	143.0	142.6	0.28	0.863	0.371	0.144
** Total carbon dioxide, mmol/L**	27.3^a^	25.6^b^	26.5^ab^	0.34	0.452	<0.001	0.162
**Urine pH**	6.96^a^	6.41^b^	6.53^b^	0.128	0.745	0.001	0.904
**Piglet immunocrit ratio^3^**	0.070	0.074	0.080	0.0034	0.430	0.068	0.406

a-c Means within row with different superscripts differ (*P* < 0.05).

1 A total of 933 mixed-parity sows (Line 1050, PIC, Hendersonville TN) and litters were used from the time of entry into the farrowing house (approximately d 112 of gestation) until cross-fostering at approximately 24 h after farrowing.

2 Farrowing protocol consisted of a control with sows receiving no intervention (None), 25 g of a Ca chloride based product (TRIAD, Alltech, Lexington, KY) top-dressed on the morning feeding of each sow from entry into the farrowing house until each sow farrowed (Calcium chloride), and a Ca gluconate protocol in which primiparous sows received a 15 mL injection and multiparous sows received a 20 mL injection of Ca gluconate if a litter had more than 16 pigs, it had been longer than 1 h since the birth of the last pig, the litter had 2 or more stillbirths, or farrowing duration exceeded 4 h (Calcium gluconate).

3 Blood was taken from one of the first 5 pigs born in each litter at approximately 24 h after the start of farrowing.

### At-risk sows

In at-risk sows, percentage of stillbirths decreased (*P* = 0.004) in CaG sows compared to control and CaCl_2_ sows (Table 3). This led to an increase (*P* = 0.006) in percentage born alive in CaG sows compared to control and CaCl_2_ sows. The percentage of sows sleeved, the number of times sows were sleeved, and WEI interval did not differ based on Ca administration protocol. Birth to cross-foster mortality decreased (*P* = 0.006) in CaG sows compared to CaCl_2_ sows with control sows intermediate. Blood Cl concentration increased (*P* = 0.039) in CaCl_2_ sows compared to control and CaG sows. Blood glucose concentration increased (*P* = 0.005) in CaG sows compared to control and CaCl_2_ sows. Circulating iCa concentration increased (*P* = 0.004) and total CO_2_ concentration in the blood decreased (*P* = 0.011) in CaCl_2_ sows compared to control sows with CaG sows intermediate.

**Table 3.** Effect of farrowing calcium protocol on sow performance for at-risk and other sows.^1^^,^^2^

	At-risk sows^3^					Other sows^4^				
Farrowing protocol:	None	Calcium chloride	Calcium gluconate	SEM	Treatment, *P* =	None	Calcium chloride	Calcium gluconate	SEM	Treatment, *P* =
**Count, *n***	139	128	144	—	—	172	184	166	—	—
**Sow entry BW, kg^5^ ^,^ ^6^**	271.1	272.2	272.0	4.66	0.953	267.3	268.1	265.8	3.13	0.738
**Gestation length, d**	116.1	116.2	116.1	0.13	0.643	116.0	115.9	115.9	0.11	0.643
**Litter characteristics**										
** Total born, *n***	16.9	16.9	17.6	0.44	0.559	16.6	16.6	16.4	0.35	0.869
** Total born alive, *n***	14.9	15.0	16.1	0.42	0.181	15.5	15.3	14.8	0.34	0.655
** Stillborn, *n* ^5^ ^,^ ^6^**	1.5^a^	1.4^ab^	1.0^b^	0.16	0.039	0.8	0.9	1.0	0.18	0.865
** Mummy, *n***	0.4	0.5	0.4	0.09	0.688	0.4	0.5	0.5	0.06	0.197
** Born alive, %^6^**	88.2^b^	88.6^b^	92.1^a^	0.98	0.006	92.9	91.3	91.0	1.08	0.163
** Stillborn, %^5^ ^,^ ^6^**	9.0^a^	8.3^a^	5.9^b^	0.91	0.004	4.8	5.6	6.1	1.09	0.772
** Mummy, %^7^**	2.6	3.0	2.1	0.48	0.670	2.4	2.9	2.9	0.34	0.102
**Farrowing characteristics**										
** Percentage of sows sleeved**	78.2	78.6	87.6	4.33	0.594	16.2	30.0	19.4	4.01	0.696
** Number of times sleeved**	2.3	2.5	2.6	0.32	0.948	0.2	0.4	0.3	0.07	0.363
** Farrowing duration, min^8^**	307	307	328	31.2	0.628	277	284	—-	23.8	0.763
**Birth-cross foster mortality, %^6^**	8.1^ab^	10.1^a^	7.6^b^	0.80	0.006	4.1^b^	6.8^a^	6.7^a^	0.54	0.003
**Wean to estrus interval, d**	5.4	5.0	5.1	0.53	0.733	4.6	5.6	4.9	0.47	0.152

a-c Means within row with different superscripts differ (*P* < 0.05).

1 A total of 933 mixed-parity sows (Line 1050, PIC, Hendersonville TN) and litters were used from the time of entry into the farrowing house (approximately d 112 of gestation) until cross-fostering at approximately 24 h after farrowing.

2 Farrowing protocol consisted of a control with sows receiving no intervention (None), 25 g of a Ca chloride based product (TRIAD, Alltech, Lexington, KY) top-dressed each morning from entry into the farrowing house until each sow farrowed (Calcium chloride), and a Ca gluconate protocol in which at-risk primiparous sows received a 15 mL injection and at-risk multiparous sows received a 20 mL injection of Ca gluconate.

3 At-risk sows (*n* = 411) were those that met the criteria to receive Ca gluconate injection (sows with > 16 pigs, > 1 h since the last birth, ≥ 2 stillbirths, or farrowing lasting > 4 h) and farrowed during working hours (6:00 a.m. until 3:00 p.m.). Because sows were assigned to treatment prior to farrowing, there were also sows assigned to the calcium gluconate treatment that were not later classified as at-risk sows and, thus, did not receive an injection of Ca gluconate.

4 Other sows included those that farrowed during working hours (6:00 a.m. until 3:00 p.m.) but did not meet the criteria for a Ca gluconate injection, as well as those that farrowed overnight when farrowing intervention was unavailable.

5 At-risk sows: Parity, *P* < 0.05.

6 Other sows: Parity, *P* < 0.05.

7 At-risk sows: Parity, *P* < 0.10.

8 Farrowing duration was collected on 79 Control sows, 84 Calcium chloride sows, and 85 Calcium gluconate sows.

### Other sows

Litter characteristics, including total born, percentage born alive, percentage stillborn, and percentage of mummies did not differ based on Ca administration protocol in all other sows. Calcium administration protocol also did not affect percentage of sows sleeved, the number of times sows were sleeved, farrowing duration, or WEI interval in these sows. Birth to cross-foster mortality was increased (*P* = 0.003) in CaG and CaCl_2_ sows compared to control sows. Blood Cl, iCa, and tCO_2_ concentrations were greater (*P* ≤ 0.044) in CaCl_2_ sows compared to control sows (Table 4). Blood Na concentration tended to be greater (*P* = 0.054) in CaCl_2_ sows compared to control sows.

**Table 4.** Effect of farrowing calcium protocol on sow blood parameters and piglet immunocrit ratio for at-risk and other sows.^1^^,^^2^

	At-risk sows^3^					Other sows^4^				
Farrowing protocol:	None	Calcium chloride	Calcium gluconate	SEM	Treatment, *P* =	None	Calcium chloride	Calcium gluconate	SEM	Treatment, *P* =
**Count, *n***	48	26	74	—	—	26	48	0	—	—
**Blood characteristics**										
** Anion gap, mmol/L**	17.7	18.0	18.2	0.49	0.428	17.4	18.1	—	0.53	0.219
** Blood urea nitrogen, mg/dL**	12.6	12.7	13.6	1.20	0.411	12.8	12.8	—	1.15	0.989
** Chloride, mmol/L**	102.9^b^	104.9^a^	103.1^b^	0.72	0.039	102.8	104.7	—	0.70	0.007
** Creatinine mg/dL^5^ ^,^ ^6^**	3.2	3.3	3.2	0.13	0.809	3.1	3.1	—	0.15	0.701
** Glucose, mmol/L**	95.6^b^	90.7^b^	102.7^a^	4.14	0.005	98.5	97.8	—	4.72	0.882
** Hematocrit, % PCV**	31.0	33.3	31.7	1.05	0.168	30.8	32.3	—	1.18	0.205
** Hemoglobin, g/dL**	10.6	11.3	10.8	0.36	0.178	10.5	11.0	—	0.40	0.212
** Ionized calcium, mmol/L^5^ ^,^ ^6^**	1.22^b^	1.29^a^	1.24^ab^	0.020	0.004	1.25	1.29	—	0.019	0.044
** Potassium, mmol/L^7^**	4.2	4.2	4.1	0.09	0.637	4.2	4.2	—	0.08	0.647
** Sodium, mmol/L^7^**	142.8	143.2	142.5	0.54	0.443	142.1	143.1	—	0.53	0.054
** Total carbon dioxide, mmol/L**	27.6^a^	25.5^b^	26.5^ab^	0.65	0.011	27.1	25.6	—	0.73	0.026
**Piglet immunocrit ratio^8^**	0.072	0.073	0.080	0.0057	0.206	0.063	0.074	—	0.0078	0.131

a-c Means within row with different superscripts differ (*P* < 0.05).

1 A total of 933 mixed-parity sows (Line 1050, PIC, Hendersonville TN) and litters were used from the time of entry into the farrowing house (approximately d 112 of gestation) until cross-fostering at approximately 24 h after farrowing.

2 Farrowing protocol consisted of a control with sows receiving no intervention (None), 25 g of a Ca chloride based product (TRIAD, Alltech, Lexington, KY) top-dressed each morning from entry into the farrowing house until each sow farrowed (Calcium chloride), and a Ca gluconate protocol in which at-risk primiparous sows received a 15 mL injection and at-risk multiparous sows received a 20 mL injection of Ca gluconate.

3 At-risk sows (*n* = 411) were those that met the criteria to receive Ca gluconate injection (sows with > 16 pigs, > 1 h since the last birth, ≥ 2 stillbirths, or farrowing lasting > 4 h) and farrowed during working hours (6:00 a.m. until 3:00 p.m.). Because sows were assigned to treatment prior to farrowing, there were also sows assigned to the calcium gluconate treatment that were not later classified as at-risk sows and, thus, did not receive an injection of Ca gluconate.

4 Other sows included those that farrowed during working hours (6:00 a.m. until 3:00 p.m.) but did not meet the criteria for a Ca gluconate injection, as well as those that farrowed overnight when farrowing intervention was unavailable.

5 At-risk sows: Parity, *P* < 0.05.

6 Other sows: Parity, *P* < 0.05.

7 At-risk sows: Parity, *P* < 0.10.

8 Blood was taken from one of the first 5 pigs born in each litter at approximately 24 h after the start of farrowing.

### Parity effects

As expected, parity 5+ sows were heavier (*P* < 0.001) at entry in the farrowing house compared to parity 2 to 4 sows; similarly, parity 2 to 4 sows were heavier than parity 1 sows at entry into the farrowing house (Table S2). These differences in entry body weight (BW) due to parity category were also found when sows were divided into at-risk and other sow categories (*P* < 0.001). Parity 5+ sows had decreased (*P* < 0.001) percentage of pigs born alive and greater (*P* < 0.001) percentage of stillbirths compared to parity 2 to 4 sows and parity 1 sows. Similarly, in both at-risk and other sows, stillbirths were greatest (*P* ≤ 0.012) in parity 5+ sows and, in sows classified as other, parity 5+ sows had a lower percentage of pigs born alive (*P* = 0.008) compared to parity 2 to 4 and parity 1 sows. Parity 5+ sows were sleeved at the greatest frequency (*P* < 0.001), followed by parity 2 to 4 sows, and then by parity 1 sows. Birth to cross-foster mortality was lower (*P* < 0.001) in parity 1 sows compared to parity 2 to 4 sows and parity 5+ sows. A similar relationship between parity and mortality was seen in birth to cross-foster mortality in sows classified as other (*P* = 0.007). There was a tendency for a difference (*P* = 0.053) in WEI based on parity category, where parity 5+ sows had a numeric decrease in WEI compared to parity 2 to 4 sows and parity 1 sows. Blood creatinine concentration was greater (*P* = 0.001) in parity 5+ and parity 2 to 4 sows compared to parity 1 sows (Table S3). A similar relationship between parity and blood creatinine concentration (*P* < 0.001) was found in sows classified as other; however, in at-risk sows, blood creatinine concentration was greater (*P* = 0.001) only in parity 5+ sows compared to parity 2 to 4 and parity 1 sows. There was a tendency for an effect (*P* = 0.059) of parity category on blood glucose level where glucose concentration was numerically greatest in parity 1 sows and decreased as parity increased. Circulating iCa concentration was decreased (*P* = 0.001) in parity 5+ sows compared to parity 1 and parity 2 to 4 sows. A similar relationship between parity and circulating iCa concentration (*P* ≤ 0.028) was seen in both at-risk and other sows. Blood K concentration tended to be different (*P* = 0.058) based on parity where parity 2 to 4 sows had a numeric decrease in K concentration compared to parity 1 and parity 5+ sows. A similar trend (*P* = 0.092) in blood K concentration based on parity was seen in at-risk sows. In at-risk sows, blood Na concentration tended to be different (*P* = 0.066) based on parity where parity 1 sows had a numeric increase in Na concentration compared to parity 2 to 4 and parity 5+ sows.

## Discussion

Stillbirths have been observed in over 48% of litters (Vanderhaeghe et al. 2010) and account for 6.9 to 8.8% of the total pigs born in the United States (MetaFarms 2024). Documented risk factors for increased stillbirth incidence include parity, farrowing duration, litter size, birth order, and level of supervision (Le Cozler et al. 2002). Fifth parity or older sows have 1.6 times greater odds of stillbirth compared to younger sows (Borges et al. 2005). This parity effect was also observed in the data herein and is likely due to reduced muscle tone affecting muscle contraction and pig expulsion (Vanderhaeghe et al. 2013). Inefficient expulsion prolongs farrowing duration, and sows that take more than 3 h to farrow have twice the odds of stillbirth (Borges et al. 2005). Larger litters (16 pigs or more) are associated with longer farrowing durations, greater maternal fatigue, and greater stillbirth incidence, particularly in the last quartile of pigs born in the litter (Adi et al. 2024). Due to the high littersize of the sows in this study, the average farrowing duration (304 min) exceeded the 3-h threshold reported by Borges et al. (2005), and the average litter size (16.8 pigs) was larger than the high-risk threshold identified by Adi et al. (2024). Consequently, the average sow in this study was at elevated risk for stillbirths.

One proposed explanation for the increased incidence of stillbirths is inadequate Ca availability. Smooth muscle contraction, including uterine contraction, is regulated by both cytosolic Ca concentration and the sensitivity of myofilaments to Ca (Uehata et al. 1997). The phosphorylation of myosin in thick filaments, which triggers smooth muscle contraction, is Ca dependent (Carsten and Miller 1987). Inadequate Ca availability for normal physiological function is referred to as hypocalcemia and has been extensively documented in dairy cows (Reinhardt et al. 2011). Hypocalcemia results from inefficient adaptation to the abrupt increase in Ca demand for milk production (Martín-Tereso and Martens 2014) which explains its greater incidence in cows with increased milk yield. In dairy cows, hypocalcemia can be classified as clinical or subclinical. Clinical cases involve overt disruptions in Ca homeostasis and are characterized by loss of appetite, lethargy, and in severe cases, recumbency (Arechiga-Flores et al. 2022). More commonly, subclinical hypocalcemia occurs when Ca levels are marginally low, without obvious clinical signs, yet this condition still impairs muscle contraction and immune function (Arechiga-Flores et al. 2022). Blim et al. (2022) observed that sows experiencing dystocia had reduced blood Ca concentrations before parturition, suggesting that similar subclinical disruptions in Ca metabolism may be occurring in sows.

One of the Ca supplements evaluated to reduce stillbirths in sows is CaCl_2_ which is an acidogenic agent that causes metabolic acidosis. This compound is added to sow diets to cause a shift in electrolyte composition which decreases the DCAD or the difference between strong positively charged cations (e.g., Na and K) and strong negatively charged anions (e.g., Cl and S). A negative DCAD results in acidification of the blood which restores tissue responsiveness to parathyroid hormone (Goff and Horst 1998). Parathyroid hormone increases Ca resorption from bone and Ca absorption in the small intestine (Goltzman 2018). A second Ca supplement, calcium gluconate, is a Ca salt of gluconic acid. This compound is given at a rate of 500 mL in dairy cows either intravenously or subcutaneously as the primary means of treating hypocalcemia (Oetzel 2022). While it has been suggested in case studies that Ca gluconate can improve farrowing kinetics and hypocalcemia in sows (Durrell 1942; Chutia et al. 2018; Reshma et al. 2020), there are currently no controlled studies that investigate it’s impact on farrowing performance.

The key production indicator targeted in this study was the incidence of stillbirths. Previous research with CaCl_2_ has led to variable results in stillbirth incidence. In general, prior research has indicated reductions in stillbirths with CaCl_2_ administration (Elrod et al. 2015; Bents and Soto 2023; González-Sánchez et al. 2023). However, Ruampatana et al. (2025) observed that CaCl_2_ did not affect stillbirth rates in supervised farrowings, but reduced stillbirth rate in sows that farrowed unsupervised. Another study reported a treatment by parity interaction, where mid-parity sows (parity 2 to 5) experienced reduced stillbirth rates with CaCl_2_ supplementation, while parity 6 or greater sows showed a numeric increase in stillbirths when provided CaCl_2_ (DeRouchey et al. 2005). Similar to the present findings, Craig et al. (2024) observed no differences in stillbirth incidence with a CaCl_2_ top-dress. Variability in the efficacy of CaCl_2_ in reducing stillbirths may be partially explained by differences in baseline stillbirth rates, duration of supplementation, or supplementation dose. In the current study, all 3 treatments exhibited lower stillbirth rates than the control treatments observed by Bents and Soto (2023), González-Sánchez et al. (2023), and Ruampatana et al. (2025). The duration of CaCl_2_ supplementation has varied across studies, with the longest reported by González-Sánchez et al. (2023) at 7 d while Bents and Soto (2023), similar to the present study, initiated supplementation around d 112 of gestation. The dosage of CaCl_2_ has also differed among studies, ranging from 25 g/d (Ruampatana et al. 2025) to 70 g/d (Elrod et al. 2015). In a pilot study by Craig et al. (2024), feed refusals were observed at doses of 80 g/d and 140 g/d, but not at 40 g/d, which was then subsequently used in their main experiment.

To our knowledge, this is the first prospective study to evaluate the effect of Ca gluconate injection on stillbirths. Because the CaG injection was administered during the peak of natural oxytocin release in farrowing (Van Den Bosch et al. 2023), it was very low stress. Although there was no statistical difference in stillbirths across the overall population, at-risk sows experienced a reduction in stillbirths following CaG injection. It is important to note that only sows meeting at-risk criteria and farrowing during normal working hours were included in this subgroup because they were the sows that were eligible to receive CaG. The numeric increase in stillbirths among other sows allotted to the CaG treatment appears to be driven by greater stillbirth rates during overnight farrowings (unpublished observations). This may have contributed to the lack of overall population differences for the CaG injection protocol.

Because the incidence of stillbirths is often associated with prolonged farrowing durations, the current study evaluated farrowing duration and farrowing assistance criteria. Elrod et al. (2015) observed that sows provided CaCl_2_ had shorter farrowing durations and improved farrowing ease scores which is indicative of reduced need for sleeving and manual pig removal. Ruampatana et al. (2025) observed no change in farrowing duration with CaCl_2_ supplementation but reported a reduction in farrowing assistance required for the first 7 pigs born. The absence of differences in farrowing duration between the control and CaCl_2_ treatments in the current study may help explain the similar stillbirth rates observed across these groups. Notably, at-risk sows injected with CaG had a numeric increase in farrowing duration but still had a lower incidence of stillbirths compared to at-risk control and CaCl_2_ sows which suggests that factors beyond farrowing duration also play a critical role in determining stillbirth outcomes.

Unlike previous studies that collected blood and urine samples from sows before farrowing, the study herein collected blood and urine post-farrowing to standardize timing across treatments, as CaG sows received injections during farrowing. In agreement with previous findings in dairy cows, CaG administration in the current study led to a reduction in urine pH (Blanc et al. 2014). Similarly, prior studies in sows have reported reduced urine pH after CaCl_2_ supplementation (DeRouchey et al. 2003; Bents and Soto 2023; Craig et al. 2024), consistent with the present results. Urine pH is reduced because the body increases the excretion of hydrogen ions (H^+^) in response to the increased dietary load of anions (such as chloride or the gluconate from calcium gluconate), as part of the kidneys’ effort to maintain acid-base balance (Darriet et al. 2017).

One of the key metabolites assessed was ionized Ca (iCa), as it is the only biologically active form of Ca (Arechiga-Flores et al. 2022). Although this is the first controlled trial to examine the effects of CaG injection on farrowing performance, Hintz and Billing (2013) investigated post-farrowing CaG administration (20 mL IM, 4 h after the completion of farrowing) as a potential strategy to mitigate hypocalcemia and reported no changes in total serum Ca, similar to the lack of differences in iCa in the current study. These results also align with findings from Craig et al. (2024) and Ruampatana et al. (2025), where no differences in blood Ca were observed with a CaCl_2_ top-dress, which may be due to the studies collecting total serum Ca instead of iCa. In contrast, both DeRouchey et al. (2003) and the present study found increased iCa and circulating Cl after CaCl_2_ administration. DeRouchey et al. (2003) speculated that the increase in iCa was due to the increase in bone mobilization needed as a buffer in the extracellular fluid due to the acidification of the blood. In addition, DeRouchey et al. (2003) observed a reduction in tCO_2_ when CaCl_2_ was added to the diet, which is similar to CaCl_2_ sows in the present study. Martins et al. (2016) documented a similar impact on tCO_2_ in dairy cattle fed acidogenic diets and speculated that it may reflect compensatory changes in respiration rate to maintain blood pH homeostasis.

Emerging evidence suggests that certain physiological criteria may serve as predictors of farrowing success. Craig et al. (2024) reported a reduced incidence of stillbirths in sows with serum Ca concentrations above 2.5 mmol/L and urine pH below 7.0. Additionally, Feyera et al. (2018) observed that greater arterial glucose levels 1 h after the birth of the first pig was associated with shorter farrowing durations. In the present study, the only difference in sow metabolites in at-risk CaG sows when compared to at-risk control and CaCl_2_ sows was elevated circulating glucose. This increase in glucose may be a key factor contributing to the reduction of stillbirths observed in at-risk CaG sows. However, the mechanism behind this rise in glucose is not fully understood. After administration, CaG dissociates into iCa and gluconic acid, but gluconic acid is not directly converted back to glucose in the blood. One possible explanation for the increase in glucose in CaG sows is that the rapid availability of Ca after the injection may have stimulated reactive tissues or endocrine responses, such as catecholamine release or altered insulin signaling, leading to a transient hyperglycemic state. Supporting this, Schlumbohm and Harmeyer (2003) demonstrated that hypocalcemia reduces endogenous glucose production. Previous work in dairy cattle has shown increased blood glucose concentrations 15 min after parturition in cows that received an oral bolus of CaCl_2_ and CaSO_4_ at the time of parturition; however, no differences in blood glucose levels were seen at the next measurement, taken 6 h after parturition (Wilms et al. 2022). It is important to note that the type of calcium supplement, route of administration, and species differ, so these results may not be directly comparable. To the best of the authors’ knowledge, no studies to date have evaluated the short-term impacts of CaG injection on glucose levels. Further research is warranted to explore the underlying mechanisms and their implications for farrowing outcomes.

In addition to increasing the risk of stillbirths, prolonged farrowing is associated with greater incidence of asphyxiated newborn pigs. Asphyxiated pigs consume less colostrum, which increases their susceptibility to hypothermia and starvation (Trujillo-Ortega et al. 2007). Based on this, we hypothesized that Ca supplementation would improve neonatal viability, thereby enhancing colostrum intake. The immunocrit procedure is an estimation of immunoglobulins in the blood which can be indicative of colostrum consumed. Interestingly, Ruampatana et al. (2025) observed that despite signs of reduced asphyxia such as lower rates of meconium staining and broken umbilical cords, colostrum intake was lower in pigs from CaCl_2_ sows compared to pigs from control sows. In the current trial, pigs from CaG sows had the greatest immunocrit which may signify improved viability and activity. However, further research is needed to fully understand the mechanism behind this improvement in immunocrit levels.

Given that farrowing duration can influence the incidence of starvation and hypothermia, it is also associated with increased early lactation pig mortality due to crushing. This is the first controlled study to evaluate the impact of Ca administration protocols on mortality of pigs in the first 24 h after farrowing. While Bents and Soto (2023) observed no differences in farrow to weaning mortality with CaCl_2_ supplementation, use of the same CaCl_2_ product in the present study resulted in increased birth to cross foster mortality, particularly in parity 1 sows, when CaCl_2_ was top-dressed. However, the authors of the current study believe that the interaction between Ca administration protocol and parity category may be an artifact, as it appears to be driven by an unusually low birth to cross-foster mortality in parity 1 control sows.

Lastly, long farrowing durations have been linked to long WEI (Oliviero et al. 2013). Therefore, we hypothesized that Ca administration protocols could influence WEI. However, consistent with findings by DeRouchey et al. (2003) and Ruampatana et al. (2025), no differences in WEI were observed in the current trial among the treatment groups.

In conclusion, for the overall sow population, topdressing the CaCl_2_-based product before farrowing or administering CaG injections peripartum affected sow blood and urine metabolites during farrowing but did not influence farrowing performance. However, when the sow population was further divided into at-risk (sows with >16 pigs, >1 h since the last birth, ≥ 2 stillbirths, or farrowing lasting > 4 h) and other sow categories, administration of a CaG injection decreased stillbirth rate in at-risk sows leading to an increase in the percentage of pigs born alive.

## Supplementary Material

txaf142_Supplementary_Data

## List of Abbreviations:

BW: body weight
CaCl_2_: calcium chloride
CaG: calcium gluconate
DCAD: dietary cation-anion difference
PWM: pre-weaning mortality
WEI: wean-to-estrus interval
