# Supplementary material for: Evaluating peripartum calcium administration strategies to improve sow farrowing performance and piglet livability
Source: Transl Anim Sci. 2025 Oct 23;9:txaf142. doi: 10.1093/tas/txaf142 (PMC12701389; doi:10.1093/tas/txaf142)
Supplement: txaf142_Supplementary_Data [file txaf142_supplementary_data.zip › R1_Ca_JBS_Supplemental_Tables_10_3_2025.docx]

| **Supplemental Table 1**. Interactive effects of farrowing calcium protocol and parity on sow farrowing performance^1^ | | | | | | | | | | | | | | | |
| --- | --- | --- | --- | --- | --- | --- | --- | --- | --- | --- | --- | --- | --- | --- | --- |
|  | Farrowing protocol^2^ | | | | | | | | | | |  |  | | |
|  | None | | |  | Calcium chloride | | |  | Calcium gluconate | | |  | *P* = | | |
| Item Parity Category^3^: | 1 | 2 to 4 | 5+ |  | 1 | 2 to 4 | 5+ |  | 1 | 2 to 4 | 5+ | SEM | Treatment × parity | Treatment | Parity |
| Count, n | 65 | 163 | 83 |  | 65 | 164 | 83 |  | 64 | 162 | 84 | --- | --- | --- | --- |
| Mummy, % | 2.0 | 3.0 | 2.7 |  | 3.6 | 3.0 | 2.9 |  | 2.2 | 2.1 | 3.7 | 0.56 | 0.019 | 0.035 | 0.228 |
| Birth-cross foster mortality, % | 3.9^b^ | 6.9^a^ | 7.8^a^ |  | 8.0^a^ | 7.4^a^ | 8.3^a^ |  | 6.1^ab^ | 7.2^a^ | 8.2^a^ | 0.85 | 0.035 | < 0.001 | < 0.001 |
| ^a-c^Means within row with different superscripts differ (*P* < 0.05).  ^1^A total of 933 mixed-parity sows (Line 1050, PIC, Hendersonville TN) and litters were used from the time of entry into the farrowing house (approximately d 112 of gestation) until cross-fostering at approximately 24 h after farrowing.  ^2^Farrowing protocol consisted of a control with sows receiving no intervention (None), 25 g of a Ca chloride based product (TRIAD, Alltech, Lexington, KY) top-dressed on the morning feeding of each sow from entry into the farrowing house until each sow farrowed (Calcium chloride), and a Ca gluconate protocol in which primiparous sows received a 15 mL injection and multiparous sows received a 20 mL injection of Ca gluconate if a litter had more than 16 pigs, it had been longer than 1 h since the birth of the last pig, the litter had 2 or more stillbirths, or farrowing duration exceeded 4 h (Calcium gluconate).  ^3^Parity was included as a fixed effect in the data analysis model. Sows were classified as P1 (n = 194), P2 to P4 (n = 489), or P5+ (n = 250). | | | | | | | | | | | | | | | |

| **Supplemental Table S2**: Effect of parity on sow farrowing performance^1,2^ | | | | | | |
| --- | --- | --- | --- | --- | --- | --- |
| Parity Category^3^ | P1 | P2-P4 | P5+ | SEM | *P* = |  |
| Count, n | 194 | 489 | 250 | --- | --- |  |
| Sow entry BW, kg | 230.4^c^ | 267.6^b^ | 309.7^a^ | 2.98 | < 0.001 |  |
| Gestation length, d | 116.0 | 116.1 | 116.1 | 0.12 | 0.405 |  |
| Litter characteristics |  |  |  |  |  |  |
| Total born, n | 17.2 | 16.6 | 16.6 | 0.30 | 0.550 |  |
| Born alive, % | 92.7^a^ | 92.3^a^ | 87.4^b^ | 1.03 | < 0.001 |  |
| Stillborn, % | 4.9^b^ | 5.0^b^ | 9.4^a^ | 1.13 | < 0.001 |  |
| Mummy, % | 2.5 | 2.6 | 3.1 | 0.27 | 0.216 |  |
| Farrowing characteristics |  |  |  |  |  |  |
| Percentage of females sleeved | 33.4^c^ | 53.3^b^ | 68.1^a^ | 3.81 | < 0.001 |  |
| Number of times sleeved | 0.6 | 1.1 | 1.9 | 0.56 | 0.359 |  |
| Farrowing duration, min | 282.3 | 316.5 | 313.7 | 23.79 | 0.350 |  |
| Birth-cross foster mortality, % | 5.8^b^ | 7.1^a^ | 8.1^a^ | 0.45 | < 0.001 |  |
| Wean to estrus interval, d | 5.7 | 5.0 | 4.5 | 0.39 | 0.071 |  |
| ^a-c^Means within row with different superscripts differ (*P* < 0.05).  ^1^A total of 933 mixed-parity sows (Line 1050, PIC, Hendersonville TN) and litters were used from the time of entry into the farrowing house (approximately d 112 of gestation) until cross-fostering at approximately 24 h after farrowing.  ^2^Farrowing protocol consisted of a control with sows receiving no intervention (None), 25 g of a Ca chloride based product (TRIAD, Alltech, Lexington, KY) top-dressed on the morning feeding of each sow from entry into the farrowing house until each sow farrowed (Calcium chloride), and a Ca gluconate protocol in which primiparous sows received a 15 mL injection and multiparous sows received a 20 mL injection of Ca gluconate if a litter had more than 16 pigs, it had been longer than 1 h since the birth of the last pig, the litter had 2 or more stillbirths, or farrowing duration exceeded 4 h (Calcium gluconate).  ^3^Parity was included as a fixed effect in the data analysis model. Sows were classified as P1 (n=194), P2-P4 (n=489), or P5+ (n=250). | | | | | |  |

| **Supplemental Table S3**: Effect of parity on sow and piglet blood and urine characteristics^1,2^ | | | | | |
| --- | --- | --- | --- | --- | --- |
| Parity Category^3^ | P1 | P2-P4 | P5+ | SEM | *P* = |
| Count, n | 38 | 121 | 63 | --- | --- |
| Blood characteristics |  |  |  |  |  |
| Anion gap, mmol/L | 18.1 | 18.0 | 17.7 | 0.40 | 0.561 |
| Blood urea nitrogen, mg/dL | 13.3 | 13.4 | 12.5 | 0.90 | 0.405 |
| Chloride, mmol/L | 104.2 | 103.4 | 102.9 | 0.55 | 0.161 |
| Creatinine mg/dL | 2.8 | 3.2 | 3.4 | 0.10 | 0.001 |
| Glucose, mmol/L | 102.9 | 98.7 | 94.1 | 3.33 | 0.059 |
| Hematocrit, %PCV | 32.5 | 31.5 | 31.2 | 0.87 | 0.455 |
| Hemoglobin, g/dL | 11.1 | 10.7 | 10.6 | 0.30 | 0.455 |
| Ionized Calcium, mmol/L | 1.28^a^ | 1.27^a^ | 1.21^b^ | 0.016 | 0.001 |
| Potassium, mmol/L | 4.2 | 4.1 | 4.2 | 0.07 | 0.058 |
| Sodium, mmol/L | 143.3 | 142.6 | 142.4 | 0.40 | 0.144 |
| Total carbon dioxide, mmol/L | 26.2 | 26.2 | 27.0 | 0.53 | 0.162 |
| Urine pH | 6.58 | 6.65 | 6.67 | 0.179 | 0.904 |
| Piglet immunocrit ratio^4^ | 0.071 | 0.077 | 0.075 | 0.0053 | 0.406 |
| ^a-c^Means within row with different superscripts differ (*P* < 0.05).  ^1^A total of 933 mixed-parity sows (Line 1050, PIC, Hendersonville TN) and litters were used from the time of entry into the farrowing house (approximately d 112 of gestation) until cross-fostering at approximately 24 h after farrowing.  ^2^Farrowing protocol consisted of a control with sows receiving no intervention (None), 25 g of a Ca chloride based product (TRIAD, Alltech, Lexington, KY) top-dressed on the morning feeding of each sow from entry into the farrowing house until each sow farrowed (Calcium chloride), and a Ca gluconate protocol in which primiparous sows received a 15 mL injection and multiparous sows received a 20 mL injection of Ca gluconate if a litter had more than 16 pigs, it had been longer than 1 h since the birth of the last pig, the litter had 2 or more stillbirths, or farrowing duration exceeded 4 h (Calcium gluconate).  ^3^Parity was included as a fixed effect in the data analysis model. Sows were classified as P1 (n=194), P2-P4 (n=489), or P5+ (n=250).  ^4^Blood was taken from one of the first five pigs born in each litter at approximately 24 h after the start of farrowing. | | | | | |
